# Tactile Low Frequency Vibration in Dementia Management: A Scoping Review Protocol

**DOI:** 10.3390/ijerph18041904

**Published:** 2021-02-16

**Authors:** Elsa A. Campbell, Jiří Kantor, Lucia Kantorová, Zuzana Svobodová, Thomas Wosch

**Affiliations:** 1 Caritas Association Ettlingen, 76275 Ettlingen, Germany; 2 VIBRAC Centre for Vibroacoustic Therapy and Research, Eino Roiha Foundation, 40014 Jyväskylä, Finland; 3 Institute for Applied Social Sciences, University of Applied Sciences Würzburg-Schweinfurt (FHWS), 97070 Würzburg, Germany; thomas.wosch@fhws.de; 4 Faculty of Education, Palacky University Center for Evidence-Based Education and Arts Therapies, Palacky University, 779 00 Olomouc, Czech Republic; jiri.kantor@upol.cz (J.K.); lucia.kantorova@mail.muni.cz (L.K.); kelnarova@med.muni.cz (Z.S.); 5 Faculty of Education, Institute of Special Education Studies, Palacky University, 779 00 Olomouc, Czech Republic; 6 Faculty of Medicine, The Czech National Centre for Evidence-Based Healthcare and Knowledge Translation (Cochrane Czech Republic, Czech CEBHC: JBI Centre of Excellence, Masaryk University GRADE Centre), Masaryk University, 625 00 Brno, Czech Republic; 7 Institute of Biostatistics and Analyses, Faculty of Medicine, Masaryk University, 625 00 Brno, Czech Republic

**Keywords:** low frequency vibration, sound vibration, mechanical vibration, vibroacoustic, dementia, music interventions

## Abstract

Dementia is a growing issue in modern society. Non-pharmacological interventions such as music are suggested as the primary methods for symptom management. Therapeutic potential may also be found in sound/mechanical low frequency vibrations (LFV) that share the core characteristics of music, but these are lesser understood. The aim of the proposed scoping review is to explore the responses of persons with dementia to LFV, e.g., vibroacoustic therapy or whole-body vibration. The scoping review will follow the Joanna Briggs Institute methodology guidelines. An extensive search in BMC, CINAHL, Cochrane Central Register of Controlled Trials, EMBASE, ERIC, MEDLINE (OvidSP), Pedro, ProQuest Central, PsycINFO, Scopus, Web of Science, and grey literature sources in Clinical Trials, Current Controlled Trials, Google Scholar, and manual search of relevant journals is planned to find all relevant research papers. The paper selection, full-text assessment, and data extraction will be performed by two independent reviewers. Participants’ responses to the interventions and the experiment designs, including methodological challenges, will be analysed and compared. Results may highlight potential gaps in reporting and comparing sound and mechanical vibration approaches and promote better understanding of their potential for managing the symptoms of dementia. Furthermore, the possible relationships between LFV and music-based interventions may become clearer.

## 1. Introduction

Dementia is a broad umbrella term used to describe a range of progressive neurological disorders associated with cognitive impairment and language and problem-solving issues including Alzheimer’s disease, frontotemporal dementia, vascular dementia, and mixed dementia [1]. The annual cost of dementia care in Europe is estimated as a little over €32,500 per person [2]. In Germany, there has been a slight reduction in informal caregiving (by family or friends) and an increase in formal caregiving (care homes) [3]. 51.8% of care home residents live with dementia, which is 19 times more than in adults living at home [4]. 78% of persons with dementia have disordered behaviour [5] and 77% have at least one other health condition (hypertension, depression, coronary heart disease, stroke or diabetes) compared to 68% in the UK general population [6]. Agitation, aggression and apathy lead to a clear reduction in residents’ quality of life and increased burden on care staff [7]. Due to the nature of the disease, assessment may be complicated by increased difficulty in comprehending questions, for example, in commonly used scales like the Cornell Scale for depression in dementia [8]. Yet, as the population ages, the proportion of those with dementia will also rise [9]. It is therefore necessary to develop and prove the effectiveness of interventions applied in residential care homes for managing the behavioural and psychological symptoms of dementia and improving residents’ independence and mobility, increasing quality of life, and reducing caretaker burden.

Pharmacological intervention is typical for managing the behavioural and psychological symptoms of dementia (BPSD). Yet, according to the Lancet Commission on Dementia, medication should only be applied in severe cases and only when psychosocial intervention alone is insufficient [9]. Currently no medications are approved by the FDA in the treatment of neuropsychiatric symptoms of dementia due to their reported side-effects and potential harm, including increased mortality rate [10]. Therefore, non-pharmacological and non-invasive interventions should be developed, examples of which are music interventions and sensory interventions based on tactile low frequency stimulation. These two types of intervention are sometimes used together, referred to as vibroacoustic therapy. The underlying mechanisms of such sensory interventions are unclear; however, theories on the therapeutic mechanisms of music may be useful in understanding how persons with dementia respond to low frequency vibration. 

Therapeutic listening and other music interventions are generally referred to in the literature as music therapy although certified music therapists may not be delivering the intervention. Music therapy comprises a goal-oriented, music-based intervention within a therapeutic relationship as defined by Bruscia [11], whilst music medicine [12] more accurately describes music listening offered by other (e.g., medical) professionals. Music interventions are effective for managing BPSD ([13], [14]) i.e., disruptive behaviours [15], socio-emotional skills [15], anxiety, depression and apathy [16], and cognitive functioning [17]. Individual interventions lasting ≥12 weeks delivered by a trained music therapist have been reported as especially effective compared to group settings [18]. Larger effect sizes [17] and homogenous outcomes have been reported from individual interventions with a therapist-client relationship but not from interventions offered from a non-music therapist and without a therapeutic relationship [19]. 

In this study, we suggest that there is a close link between music and tactile low frequency stimulation. Approaches based on low frequency sound vibration are sometimes classified under receptive music therapy techniques [20,21]. However, music intervention reviews seldom include tactile low frequency stimulation methods; furthermore, the latter area is lesser understood and researched. The intention to explore the potential of tactile low frequency stimulation in people with dementia may be justified by possible similarities in the therapeutic mechanisms of both the auditory and tactile forms of music.

### 1.1. The Connection Between Music, Vibration and Cognition

Perceiving and making music involves several cognitive processes which influence attention, memory, motor action planning and communication; music also has an impact on emotion processing and regulation [22]. The elements of music, e.g., pitch, rhythm, amplitude, play an important role in how music is perceived, either through auditory perception or tactile sensation, i.e., vibration. Music is inextricably linked to vibration, since music is sound and sound and pitch relate to frequencies (Hz); indeed, music theory had traditionally been a part of mathematical subjects [23]. Tempo and rhythm are perceived in the most primitive neural centres of the brain and stimulate a thalamic reflex causing unconscious tapping to the beat; in this way these elements can be used to connect to movement (such as walking) [21]. Different brainwave frequencies are related to different perceptual, motor, or cognitive states and have also been shown to synchronise with external stimuli (e.g., auditory stimuli). Synchronisation with auditory stimuli within beta and gamma ranges is also proposed to contribute to rhythmic entrainment for cognitive functions related to learning and memory tasks [24]. Tactile stimuli (vibrations) using frequencies targeted at specific bandwidths in the brain could produce similar neural entrainment as reported from rhythmic auditory stimuli such as music, given that the underlying mechanisms are potentially similar (see Section 1.2) [25]. Due to this link between music and vibration, the impact of various oscillatory rhythms on dementia must also be considered. 

Neural rhythmic oscillatory activity has been proposed to play a role in neurodegenerative diseases and neurorehabilitation [25]. Memory processes and communication within local brain networks (e.g., sensory cortex) are proposed as happening primarily within the gamma frequency range (30–100 Hz) and between more distant brain areas at 4-8 Hz on the theta frequency range. Deep brain stimulation is used as a treatment on the premise that these circuits are dysregulated; this may be due to a lack of coherence because of inadequate or disturbed excitation. Thalamocortical dysrhythmia, when alpha oscillatory activity nears the theta band and gamma band activity reduces, is shown to be connected to motoric, mood, auditory, and cognitive functioning (e.g., Parkinson’s disease) as well as a wide range of cognition, memory, and executive functions. Frequencies lying within the 20–120 Hz range are classified as low frequency. There are various modes of delivering low frequencies such as sound vibration (vibroacoustic therapy), mechanical, whole-body vibration (WBV), or electrical approaches like deep brain stimulation (DBS), transcranial magnetic stimulation (TMS) and transcranial direct current stimulation (tDCS). Although these electrical methods are used in dementia care, the underlying mechanisms of tactile vibration are proposed to include an induced relaxation response [26] due to its massage-like sensation, induced by excitation of corpuscles under the skin receptive to specific frequencies [27]. Due to this, electrical stimulation methods are excluded from this review. Low frequency vibration may be included as a part of multisensory environment, but it is usually combined with other interventions and stimuli [28]. 

### 1.2. Description of Tactile Low Frequency Vibration Interventions

Vibroacoustic therapy (VAT) comprises tactile, low frequency, sinusoidal sound vibration (20–100 Hz) combined with music listening and a therapeutic relationship [27]. This approach incorporates the physical and auditory perception of music, i.e., the therapeutic benefits of both auditory and tactile sound. The vibration is delivered through specially designed equipment such as mattresses with in-built transducers/loudspeakers. The client may listen to their preferred music to support the pre-defined therapeutic goals. Other terms used to describe this intervention include physioacoustic therapy, vibroacoustic treatment, vibrotactile stimulation, low frequency sound stimulation, and rhythmic sensory stimulation [29]. Research has focused on the 40 Hz level, as it affords a general relaxation response [30]. Studies in this area have been mostly non-controlled or case studies/reports and have implemented questionnaires such as the Mini Mental State Examination or Saint Louis University Mental Status (e.g., [31]). 

Whole body vibration (WBV) is another tactile vibration method sometimes used in conjunction with exercise/physiotherapy, in which persons stand on an oscillating platform (either performing exercises or passively receiving the stimulation). It has been shown to be beneficial for improving balance and mobility in institutionalised elderly persons and in general health [32], as well as for cognitive functioning [33]. These studies have included RCTs and have utilised questionnaires as well as physiological measures such as blood circulation and bone metabolism [34]. It is suggested that WBV may mitigate the ageing process in musculoskeletal structures [35], but the optimal frequency, amplitude and level of muscle activation should be identified. Although both VAT and WBV utilise tactile sinusoidal vibration, the characteristics of these interventions and their outcomes for dementia symptom management have not been compared. 

### 1.3. Aim of the Scoping Review

Currently, there is no overview of the characteristics of tactile low frequency sound or mechanical vibration used for managing the symptoms of dementia, which represents a gap in knowledge. Because fewer results are expected and there are potentially valuable reports from clinical practice and non-research papers, a scoping review design was deemed more appropriate than a systematic review with meta-analysis, focusing on precise investigations of the effectiveness of an intervention using specific outcomes. Furthermore, the aim of this review is to identify the available evidence, highlight and analyse the gaps in current knowledge, delineate the key aspects and definitions in the literature and report on how the research has been conducted [36]. In the case of mapping and discussing intervention characteristics or concepts and their evidence sources, the Joanna Briggs Institute (JBI) suggests the scoping review as the more appropriate design [37]. 

Clinicians and researchers planning to design and implement future studies may benefit from a summary of how low frequency vibration is used for therapeutic purposes within and outside the context of music-based interventions, especially given the Lancet Commission Report’s findings [9]. Moreover, future researchers may benefit from a discussion of the methodological aspects that can be impacted by clinical characteristics of dementia, e.g., concerning the appropriate research designs, control group sham interventions, or data collection methods and outcome measures.

The aim of this scoping review is to summarise the research on tactile low frequency sound and mechanical vibration approaches that have been carried out with adults with dementia. We are interested in participants’ responses to, and the properties of, the interventions. This includes, for example, how participant responses have been collected and analysed (e.g., Neuropsychiatric Inventory Scale or neuroimaging). We are also interested in how methodological challenges related to the clinical aspects of dementia and how issues in applying vibration as a therapeutic medium were solved by different authors. Based on the critical appraisal of the studies and exploration of the research experiments, we will make recommendations for future studies. In the proposed scoping review, we will not limit the search strategy to a particular type of research or study design, with the intention of obtaining an inclusive map of the field. Therefore, the scoping review design was chosen as the most appropriate for these purposes [38].

Furthermore, we want to address interdisciplinary questions concerning the relationship between approaches using sound/mechanical vibration and music-based interventions. VAT is sometimes defined as being a receptive music therapy method (e.g., [21]) (although it may be applied as a stand-alone therapy) yet it is seldom discussed in literature on receptive music therapy or music medicine. This gap in knowledge supports the rationale for investigating this therapy method, along with the ever-increasing need for dementia care strategies and the Lancet Commission’s recommendation of music therapy for BPSD. 

A preliminary search in Epistemonikos, Cochrane Review, JBI Evidence Synthesis, Open Science Framework and Prospero did not return any results on scoping or systematic reviews on VAT/WBV and dementia; only systematic reviews on auditory musical interventions were found for this population. The Population, Concept, Context (PCC) paradigm was used to establish the focus of the review:

Population (P): Adults with dementia

Concept (C): Tactile low frequency sinusoidal sound or mechanical vibration

Context (C): Not limited 

These were then used to formulate the review questions:

- RQ1: What participant responses (e.g., reduced agitation) are reported in studies on tactile low frequency vibration and dementia and how have they been measured?
- RQ2: What intervention characteristics (e.g., Hz used, duration of treatment) are reported in studies on tactile low frequency vibration and dementia and how do these compare or differ across the approaches?
- RQ3: What are the specifics of the research experiments in studies on tactile low frequency vibration and dementia?

## 2. Materials and Methods

The proposed scoping review will be conducted in accordance with the JBI methodology for scoping reviews [38] and according to the extended PRISMA statement for scoping reviews [39]. 

### 2.1. Inclusion Criteria

The inclusion criteria, defined based on the PCC paradigm and the review questions, are as follows:

- Participants: Adults with all types of dementia, both genders, patients with any level of functioning. The age limit is not set, so as to include all studies reporting on adults of any age with dementia, and types of dementia affecting younger adults (i.e., Huntington’s disease). Studies of dementia patients with various comorbidities will be included if the intervention is focused on dementia.
- Concept: Studies addressing the use of tactile low frequency vibration e.g., VAT, with or without music listening and with or without a therapeutic relationship for managing the symptoms of dementia. Studies reporting on whole body vibration (WBV) or interventions using local mechanical vibrations will also be included, along with papers using these interventions in multisensory environments, if the participants’ response to low frequency vibration may be distinguished from responses to other stimuli. Studies based on electrical vibrations, e.g., transcranial magnetic stimulation, or low tone natural sounds (without technologically modified vibration) will be excluded.
- Context: There will be no limitation on demographic context or settings. We will search for studies from all over the world done in residential care homes, hospitals, private setting, etc. The search will be limited to articles written in English, German, and Czech. The language of the study will be recorded in the data extraction table.
- Types of studies: All types of quantitative, qualitative and mixed-methods studies including systematic and scoping reviews will be included. Designs of quantitative studies will include experimental studies, quasi-experimental studies, observational studies, case series/case studies, cross-sectional studies, and case reports from clinical practice. Qualitative studies will not be limited by the paradigm, although mainly phenomenological studies are expected to be found in this area.

### 2.2. Search Strategy

The search strategy will aim to locate both published and unpublished studies. An initial limited search of EMBASE, CINAHL plus and MEDLINE (Ovid PS) was undertaken to identify articles on the topic. The text words contained in the titles and abstracts of relevant articles, and the index terms used to describe the articles, were used to develop a full search strategy for the databases (see Appendix Table A1, Table A2 and Table A3). The search strategy, including all identified keywords and index terms, will be adapted for each included database and/or information source. The reference list of all included sources of evidence will be screened for additional studies. The databases to be searched include BMC (Medvik), CINAHL Plus, Cochrane Central Register of Controlled Trials, EMBASE, ERIC, MEDLINE (OvidSP), Pedro, ProQuest Central, PsycINFO, Scopus, and Web of Science. Sources of grey literature to be searched include Clinical Trials, and Current Controlled Trials, Google Scholar (first 300 hits). Furthermore, we will conduct a hand search in the reference lists of all returned articles, in all issues of journals “Voices: A World Forum for Music Therapy”, “Approaches: An Interdisciplinary Journal of Music Therapy” and “Music and Medicine”, and in the books “Music Vibration and Health” (all chapters) and “The Art and Science of Music Therapy” (chapter by Wigram and Skille). The search period will date from 1980 to the present. 

Following the search, all identified citations will be collated and uploaded into Zotero 5.0 (Roy Rosenzweig Center for History and New Media, George Mason University, Fairfax, VA, USA) duplicates removed. Following a pilot test, titles and abstracts will then be screened by two independent reviewers for assessment against the inclusion criteria for the review (EAC, JK). The full text of potentially relevant sources will be assessed in detail against the inclusion criteria by two independent reviewers. Reasons for exclusion of sources of evidence at full text that do not meet the inclusion criteria will be recorded and reported in the scoping review. Any disagreements that arise between the reviewers at each stage of the selection process will be resolved through discussion or with an additional reviewer/s (LK, TW). The results of the search and the study inclusion process will be reported in full in the final scoping review and presented in a Preferred Reporting Items for Systematic Reviews and Meta-analyses for Systematic Reviews (PRISMA-SR) flow diagram [35]. 

### 2.3. Assessment of Methodological Quality and Extraction of Data

Articles selected for retrieval will be assessed by two independent reviewers (EAC, JK) for methodological quality and, based on the character of the identified full texts, the reviewers will consider performing a full critical appraisal using standardised critical appraisal from JBI [40]. Any disagreements that arise between the reviewers will be resolved through discussion and by a third reviewer (LK).

Data will be extracted from papers included in the scoping review by two independent reviewers (EAC, JK) using a data extraction tool developed by the reviewers (see Table 1). We will extract data related to author and year of the study, country and setting, study design/research experiment description (sham, blinding, method of allocation, methodological challenges mentioned by researchers, etc.), research sample and type of dementia, as well as potential co-morbidity diagnoses, participant characteristics (age, gender, education and profession), intervention characteristics (including frequencies used, device, pulsation speed/duration, amplitude (dB), duration (time), scan (Hz) and the absence or presence of music listening as well as whether this was participant or researcher/therapist chosen). The music listened to, and primary and secondary outcomes and outcome measures, will also be extracted. The draft data extraction tool will be modified and revised as necessary during the pilot process of data extraction. Any disagreements that arise between the reviewers will be resolved through discussion or with an additional reviewer/s (LK, TW). If appropriate, authors will be contacted to request missing or additional data if required. A critical appraisal of all included literature will be conducted based on JBI´s critical appraisal tools according to the type of research study.

## 3. Results and Discussion

This section will present the data in tabular form supported by a narrative, structured according to the review’s research questions. Review question 1 will address the participant responses to the interventions and how these responses have been investigated. In the data extraction table, we plan to classify the responses according to the type of response, e.g., physiological, behavioural or psychological responses, quality of life, depression, agitation, activities of daily living, etc. Additionally, sub-group analyses will be conducted according to types of dementia, reported age groups of the participants, and intervention/approach used and whether its application was whole body or local. Outcome measures and other methods used to gather the data will be reported and classified into specific categories according to the type of measure, e.g., questionnaires or observation, and the purpose of the measure, e.g., BPSD or quality of life. The intervention characteristics and their similarities/differences across interventions will be reported in answer to Review Question 2. We expect that the studies will mention at least the brand name of the technology used, the locality of body application (local or whole body) and the frequencies (in Hz) of low frequency sound. Furthermore, all the relevant information related to the intervention and other low frequency vibration characteristics will be added and compared across the studies. In answer to Review Question 3, we will analyse the specifics of the research experiments depending on the design of the study. The JBI codes for different levels of evidence in quantitative research will be used here [41]. In qualitative studies, the research paradigm will be identified. Moreover, based on the information gathered during the critical appraisal and data extraction we will compare studies with the same type of research and design, e.g., concerning the blinding, shams used, methods of allocation, confounding factors, or length of interventions.

Furthermore, we expect to gain insight into the current gaps in the literature regarding study designs and methods as well as authors’ recommendations for future research and the limitations of these studies regarding, e.g., valid controls, suitable study designs, and the potential impact of age in designing low frequency studies. An overview of data related to the year of publication will show the research trends in this area. These results may be significant in highlighting the potential gaps in reporting (such as intervention characteristics) which could lead to more transparent reporting and a better understanding of the implications of LFV in dementia symptom management. Moreover, a better understanding of the intervention characteristics may shed light on how they may be defined, their relationship to music interventions and their potential as a non-pharmacological intervention. The limitations of these interventions, possibly related to study design, difficulty in delivering the intervention to frailer older adults, or dosage, will also be reported. 

The possible auditory music listening stimulus as part of vibroacoustic therapy sessions will also be discussed and juxtaposed with receptive music therapy and music medicine interventions to contextualise the scoping review outcomes. This affords the possibility of comparing this intervention with mechanical tactile vibration interventions within the framework of the Lancet Commission on Dementia report [9]. VAT is often viewed as a method distinct from music therapy, although Hooper [20] and Grocke and Wigram [42] have described the approach as a receptive music therapy method. The implications for vibroacoustic therapy as a stand-alone or music therapy method will also be discussed depending on the literature and the theoretical framework in which vibroacoustic therapy is presented. These results could offer a theoretical framework which would be useful when reporting further research on VAT, and potentially WBV.

## 4. Conclusions

The reported responses of patients with dementia on tactile low frequency sound and mechanical vibration will be investigated in this scoping review. We aim to gain greater understanding of interventions using low frequency vibrations and the methodological specifics and challenges of researching low frequency vibrations in people with dementia. Based on this analysis, this review could be beneficial in formulating recommendations for future research in terms of the selection of study design and outcome measures, music selection, a stricter focus on reporting the concrete effects of these stimuli, choice and transparent selection of the stimulus parameters (e.g., frequencies, amplitude) applied in research, and specifically those used with persons with dementia.

## Figures and Tables

**Table 1:** Data extraction tool developed by the authors.

Author, year Country, setting, language
Study design / research experiment description (sham, blinding, method of allocation, methodological challenges mentioned by researchers, etc.) Research sample, type of dementia (diagnosis), co-morbid/ secondary diagnosis(es)
Participant characteristics (age, gender, education, profession)
Tactile low frequency intervention characteristics (type of vibration [sound, mechanical], frequency (Hz), device, pulsation / cycle duration, amplitude (dB), duration (time), absence/presence of music listening, absence/presence of therapeutic relationship)
Music choice Participant/client or therapist chosen music listening
Outcome measures Primary outcome measures Secondary outcomes measures
Outcomes Primary outcomes Secondary outcomes

## Appendix A. Examples of Search Formula

**Table A1:** Database: CINAHL Plus with Full Text.

	Search
1	(MH “Dementia+”) (Major Headings with Subheadings)
2	(MM “Alzheimer´s Disease”) (Major Headings with Subheadings)
3	(MM “Levy Body Disease”) (Major Headings with Subheadings)
4	(MH “Huntington´s Disease”) (Major Headings with Subheadings)
5	Dementia Ti/Ab
6	Alzheimer* Ti/Ab
7	Lewy Ti/Ab
8	Klüver-Bucy syndrom* Ti/Ab
9	Kluver Ti/Ab
10	Huntington Ti/Ab
11	1–10 OR
12	Vibro-acoustic Ti/Ab
13	Physio-acoustic Ti/Ab
14	Somatron Ti/Ab
15	Low-frequency sound Ti/Ab
16	Low-frequency vibration Ti/Ab
17	Vibrotactile stimulation Ti/Ab
18	Vibro-tactile Ti/Ab
19	Rhythmic sensory stimulation Ti/Ab
20	Whole body vibration Ti/Ab
21	Vibration therapy* Ti/Ab
22	11-21 OR
23	11 AND 22

**Table A2:** Database: EMBASE.

	Search
1	‘Dementia’ / exp
2	‘Alzheimer Disease’ / exp
3	‘Kluver Bucy syndrom*’ / exp
4	‘Lewy body’ / exp
5	Dementia Ti/Ab
6	‘Huntington chorea’ Ti/Ab
7	Alzheimer* Ti/Ab
8	Kluver Ti/Ab
9	Huntington Ti/Ab
10	Lewy Ti/Ab
11	1–10 OR
12	Vibro-acoustic* Ti/Ab
13	Physio-acoustic* Ti/Ab
14	Somatron* Ti/Ab
15	Low-frequency sound* Ti/Ab
16	Low-frequency vibration Ti/Ab
17	Vibrotactile stimulation Ti/Ab
18	Vibro-tactile* Ti/Ab
19	Rhythmic sensory stimulation* Ti/Ab
20	Whole body vibration Ti/Ab
21	Vibration therapy* Ti/Ab
22	12-21 OR
23	11 AND 22

**Table A3:** Database: PubMed.

	Search
1	Dementia [Mesh]
2	Alzheimer Disease [Mesh]
3	Lewy Body Disease [Mesh]
4	Huntington Disease [Mesh]
5	Kluver-Bucy syndrome [Mesh]
6	Dementia Ti/Ab
7	Alzheimer* Ti/Ab
8	Lewy Ti/Ab
9	Kluver Ti/Ab
10	Huntington Ti/Ab
11	1–10 OR
12	Vibro-acoustic* Ti/Ab
13	Physio-acoustic* Ti/Ab
14	Somatron Ti/Ab
15	Low-frequency sound* Ti/Ab
16	Low-frequency vibration Ti/Ab
17	Vibrotactile stimulation* Ti/Ab
18	Vibro-tactile Ti/Ab
19	Rhythmic sensory stimulation* Ti/Ab
20	Whole body vibration* Ti/Ab
21	Vibration therapy* Ti/Ab
22	11–21 OR
23	11 AND 22

## References

[1] What Is Dementia? Alzheimer’s Disease and Dementia. https://alz.org/alzheimers-dementia/what-is-dementia.

[2] Cantarero-Prieto D., Leon P.L., Blazquez-Fernandez C., Juan P.S., Cobo C.S. (2019). The economic cost of dementia: A systematic review. Dementia.

[3] Ramroth H., Specht-Leible N., König H.-H., Mohrmann M., Brenner H. (2006). Use of hospital based resources by individuals in residential care homes. Dtsch. Arztebl. Int..

[4] Hoffmann F., Kaduszkiewicz H., Glaeske G., Bussche H.V.D., Koller D. (2014). Prevalence of dementia in nursing home and community-dwelling older adults in Germany. Aging Clin. Exp. Res..

[5] Seitz D., Purandare N., Conn D. (2010). Prevalence of psychiatric disorders among older adults in long-term care homes: A systematic review. Int. Psychogeriatr..

[6] Dementia: Comorbidities in Patients—Data Briefing. GOV.UK. https://www.gov.uk/government/publications/dementia-comorbidities-in-patients/dementia-comorbidities-in-patients-data-briefing.

[7] Miyamoto Y., Tachimori H., Ito H. (2010). Formal Caregiver Burden in Dementia: Impact of Behavioral and Psychological Symptoms of Dementia and Activities of Daily Living. Geriatr. Nurs..

[8] Sheehan B. (2012). Assessment scales in dementia. Ther. Adv. Neurol. Disord..

[9] Livingston G., Huntley J., Sommerlad A., Ames D., Ballard C., Banerjee S., Brayne C., Burns A., Cohen-Mansfield J., Cooper C. (2020). Dementia prevention, intervention, and care: 2020 report of the Lancet Commission. Lancet.

[10] Pharmacological Management of Neuropsychiatric Symptoms of Dementia | SpringerLink. https://link.springer.com/article/10.1007/s40501-020-00233-9.

[11] Bruscia K.E. (2014). Defining Music Therapy University Park.

[12] Dileo C. (2013). A Proposed Model For Identifying Practices: A Content Analysis of the First 4 Years of Music and Medicine. Music. Med..

[13] Tsoi K.K., Chan J.Y., Ng Y.-M., Lee M.M., Kwok T.C., Wong S.Y. (2018). Receptive Music Therapy Is More Effective than Interactive Music Therapy to Relieve Behavioral and Psychological Symptoms of Dementia: A Systematic Review and Meta-Analysis. J. Am. Med Dir. Assoc..

[14] Gómez-Romero M., Jiménez-Palomares M., Rodríguez-Mansilla J., Flores-Nieto A., Garrido-Ardila E.M., González-López-Arza M.V. (2017). Benefits of music therapy on behaviour disorders in subjects diagnosed with dementia: A systematic review. Neurol. Engl. Ed..

[15] Fakhoury N., Wilhelm N., Sobota K.F., Kroustos K.R. (2017). Impact of Music Therapy on Dementia Behaviors: A Literature Review. Consult. Pharm..

[16] Lam H.L., Li W.T.V., Laher I., Wong R.Y. (2020). Effects of Music Therapy on Patients with Dementia—A Systematic Review. Geriatrics.

[17] Chang Y.-S., Chu H., Yang C.-Y., Tsai J.-C., Chung M.-H., Liao Y.-M., Chi M.-J., Liu M.F., Chou K.-R. (2015). The efficacy of music therapy for people with dementia: A meta-analysis of randomised controlled trials. J. Clin. Nurs..

[18] Fusar-Poli L., Bieleninik Ł., Brondino N., Chen X.-J., Gold C. (2018). The effect of music therapy on cognitive functions in patients with dementia: A systematic review and meta-analysis. Aging Ment. Health.

[19] Vasionytė I., Madison G. (2013). Musical intervention for patients with dementia: A meta-analysis. J. Clin. Nurs..

[20] Hooper J. (2001). An Introduction to Vibroacoustic Therapy and an Examination of its Place in Music Therapy Practice. Br. J. Music. Ther..

[21] Grocke D., Wigram T. (2007). ‘Vibroacoustic therapy in receptive music therapy’. Recept. Methods Music Ther. Tech. Clin. Appl. Music Ther. Clin. Educ. Stud..

[22] Uhlig S., Jaschke A., Scherder E. Effects of music on emotion regulation: A systematic literature review. Proceedings of the 3rd International Conference on Music and Emotion (ICME3).

[23] Dostrovsky S. (1975). Early vibration theory: Physics and music in the seventeenth century. Arch. Hist. Exact Sci..

[24] Will U., Berg E. (2007). ‘Brain wave synchronization and entrainment to periodic acoustic stimuli’. Neurosci. Lett..

[25] Bartel L.R., Chen R., Alain C., Ross B. (2017). Vibroacoustic Stimulation and Brain Oscillation: From Basic Research to Clinical Application. Music. Med..

[26] Benson H., Klipper M.Z. (1975). The Relaxation Response.

[27] Punkanen M., Ala-Ruona E. (2012). Contemporary Vibroacoustic Therapy: Perspectives on Clinical Practice, Research, and Training. Music. Med..

[28] Lorusso L.N., Bosch S.J. (2018). Impact of multisensory environments on behavior for people with dementia: A systematic literature review. Gerontologist.

[29] Ala-Ruona E., Punkanen M. (2017). Multidisciplinary applications of vibroacoustics–from clinical practice and research to future directions. Music. Med..

[30] Campbell E.A., Hynynen J., Burger B., Vainionpää A., Ala-Ruona E. (2019). Vibroacoustic treatment to improve functioning and ability to work: A multidisciplinary approach to chronic pain rehabilitation. Disabil. Rehabil..

[31] Clements-Cortes A., Ahonen H., Evans M., Tang-Wai D., Freedman M., Bartel L. (2017). Can Rhythmic Sensory Stimulation Decrease Cognitive Decline in Alzheimer’s Disease?. Music. Med..

[32] Zhang L., Weng C., Liu M., Wang Q., Liu L., He Y. (2013). Effect of whole-body vibration exercise on mobility, balance ability and general health status in frail elderly patients: A pilot randomized controlled trial. Clin. Rehabil..

[33] Kim K.-H., Lee H.-B. (2018). The effects of whole body vibration exercise intervention on electroencephalogram activation and cognitive function in women with senile dementia. J. Exerc. Rehabil..

[34] Lam F.M.H., Liao L.R., Kwok T.C.Y., Pang M.Y.C. (2017). Effects of adding whole-body vibration to routine day activity program on physical functioning in elderly with mild or moderate dementia: A randomized controlled trial. Int. J. Geriatr. Psychiatry.

[35] Cardinale M. (2005). Whole body vibration exercise: Are vibrations good for you? * Commentary. Br. J. Sports Med..

[36] Munn Z., Peters M.D., Stern C., Tufanaru C., McArthur A., Aromataris E. (2018). Systematic review or scoping review? Guidance for authors when choosing between a systematic or scoping review approach. BMC Med. Res. Methodol..

[37] 1 Why a Scoping Review?—JBI Manual for Evidence Synthesis—JBI GLOBAL WIKI. https://wiki.jbi.global/pages/viewpage.action?pageId=3178748.

[38] Peters M.D.J., Godfrey C., McInerney P., Munn Z., Tricco A.C., Khalil H., Aromataris E., Munn Z. Chapter 11: Scoping Reviews (2020 version). In JBI Manual for Evidence Synthesis, JBI 2020. https://synthesismanual.jbi.global.

[39] Tricco A.C., Lillie E., Zarin W., O’Brien K.K., Colquhoun H., Levac D., Moher D., Peters M.D.J., Horsley T., Weeks L. (2018). PRISMA Extension for Scoping Reviews (PRISMA-ScR): Checklist and Explanation. Ann. Intern. Med..

[40] Moola S., Munn Z., Tufanaru C., Aromataris E., Sears K., Sfetc R., Currie M., Lisy K., Qureshi R., Mattis P., Aromataris E., Munn Z. (2020). Chapter 7: Systematic reviews of etiology and risk. In JBI Manual for Evidence Synthesis, Joanna Briggs Inst.

[41] Marečková J., Klugarová J. (2015). Evidence-Based Healthcare.

[42] Grocke D., Wigram T. (2007). Receptive Methods in Music Therapy: Techniques and Clinical Applications for Music Therapy Clinicians, Educators and Students.

